# The Protective Effects of Cath-MH With Anti-Propionibacterium Acnes and Anti-Inflammation Functions on Acne Vulgaris

**DOI:** 10.3389/fphar.2021.788358

**Published:** 2021-12-09

**Authors:** Jiena Wu, Ruiyin Guo, Jinwei Chai, Weichen Xiong, Maolin Tian, Wancheng Lu, Xueqing Xu

**Affiliations:** Guangdong Provincial Key Laboratory of New Drug Screening, School of Pharmaceutical Sciences, Southern Medical University, Guangzhou, China

**Keywords:** acne vulgaris, antimicrobial peptide, Microhyla heymonsivogt, Cath-MH, inflammation, lipoteichoic acid, lipopolysaccharide

## Abstract

Acne vulgaris is a common adolescent skin condition which is mainly caused by *Propionibacterium acnes* overcolonization and subsequent inflammation. Our previous studies have demonstrated that Cath-MH, an antimicrobial peptide from the skin of the frog *Microhyla heymonsivogt*, possesses potential antimicrobial, LPS-binding, and anti-septicemic properties. However, its protective effects and potential mechanisms against acne vulgaris are still unclear. In the present study, its anti-*P. acnes* effects were measured by two-fold broth dilution method, agglutination assay, scanning electron microscopy and confocal laser scanning microscopy experiments. Its treatment potential for acne vulgaris was further evaluated in mice ear inoculated by *P. acnes*. In addition, the binding ability between Cath-MH and LTA was measured by the Circular Dichroism and antibacterial assay. Moreover, the anti-inflammatory efficiency of Cath-MH was evaluated in LTA- and LPS-induced RAW 264.7 macrophage cells. Cath-MH was found to kill *P. acnes* with a MIC value of about 1.56 μM by membrane disruption mechanism. It also exhibited agglutination activity against *P. acnes*. Cath-MH was able to bind LTA as well as LPS, inhibit LTA/LPS-stimulated TLR2/4 expression, and subsequently decreased the inflammatory response in RAW 264.7 cells. As expected, Cath-MH alleviated the formation of edema and the infiltration of inflammatory cells in acne mouse model with concurrent suppression of *P. acnes* growth and inflammatory cytokines expression *in vivo*. The potent *P. acnes* inhibition activity combined with powerful anti-inflammatory effect of Cath-MH indicates its potential as a novel therapeutic option for acne vulgaris.

## Introduction

Acne vulgaris is one of the most familiar chronic inflammatory skin conditions which affects 80–85% of adolescents globally (Cong et al., 2019; Habeshian and Cohen, 2020). Although acne vulgaris is common, its etiology is still not fully understood and is believed to be multifactorial. As an opportunistic pathogen in acne vulgaris, *Propionibacterium acnes* overcolonization of the pilosebaceous follicle is considered as one of the central factors of acne vulgaris, which induces the secretion of lipase, hyaluronidase as well as proteases and activation of immune cells, thus initiating inflammation (Tanghetti, 2013; Cong et al., 2019). Besides skin pathogenic bacteria and inflammation, two other factors involved in this chronic inflammatory skin disease are the increased sebum production and follicular hyperkeratinization (Dréno, 2017). Commonly used anti-acne drugs include benzoyl peroxide, retinoids, salicylic acid, isotretinoin, antibiotics and hormonal agents. However, almost all of them have certain drawbacks like causing skin irritation, dry skin, immune hypersensitivity, organ damage, and photosensitivity (Lee et al., 2014). Specially, the long-term use of antibiotics may even induce bacterial resistance, which makes some antibiotics become ineffective for acne vulgaris (Nakase et al., 2014; Cong et al., 2019). Therefore, it is essential to develop alternative therapeutic agents with fewer adverse effects and high efficacy (Habeshian and Cohen, 2020).

Antimicrobial peptides (AMPs) are the important effectors of the innate immune system in the skin and provide the first line of defense against invading microorganisms (Woodburn et al., 2019), (Mansour et al., 2014). Typically, AMPs which are generally amphipathic and cationic can electrostatically interact with the anionic bacterial membrane and cause membranolysis (Wimley, 2010). In addition to the well-known antimicrobial properties, AMPs also have other effects like anti-inflammation, lipopolysaccharide (LPS) or/and lipoteichoic acid (LTA) binding, bacterial agglutination and so on (Heinbockel et al., 2013; Xu and Lai, 2015; Zeng et al., 2018; Ye et al., 2020). For example, cathelicidin-PY has both antimicrobial and anti-inflammatory activities, FM-CATH can not only trigger the agglutination of bacteria but also bind to LPS and LTA, and LL-37 can modulates the immune responses (Wei et al., 2013; Hancock et al., 2016; Wu et al., 2021). Compared with conventional antibiotics, AMPs are able to modulate host immune responses and less likely to cause microbial resistance in the short term due to the distinct modes of action (Zelezetsky et al., 2006). Thus, AMPs can be potential candidate for treating acne vulgaris and some of them are reported to show treatment potential for acne vulgaris by direct killing bacteria and inhibiting Toll-like receptor 2 (TLR2)-induced NF-κB activation (Marta Guarna et al., 2006).

We previously identified and characterized a novel AMP, Cath-MH, from the skin of the frog *Microhyla heymonsivogt*, which possesses single α-helical structure in membrane-mimetic environments. Cath-MH can kill fungi and bacteria, bind LPS, and inhibit LPS- as well as cecal ligation and puncture-induced sepsis through its antimicrobial, LPS-neutralizing, coagulation suppressing effects as well as suppression of MAPK signaling (Chai et al., 2021). Taking into account the bioactivities of Cath-MH, our present study is conducted to assess the anti-*P. acnes* and anti-inflammatory properties of Cath-MH *in vitro* and *in vivo*. Our results suggest that Cath-MH might be an excellent therapeutic agent for acne vulgaris.

## Materials and Methods

### Animals and Ethics Statement

All six-week-old BALB/c mice were obtained from the Laboratory Animal Center of Southern Medical University, and were reared in the SPF facility at Southern Medical University. The animal experiments were carried out in the light of the approval and guidelines of Animal Care and Use Committee of Southern Medical University. All procedures in this study strictly complied with the Animal Welfare Act and principles stated in the Guide for the Care and Use of Laboratory Animals, National Research Council, 1996.

### Peptide Synthesis

Cath-MH (APCKLGCKIKKVKQKIKQKLKAKVNAVKTVIGKISEHLG) and FITC-labeled Cath-MH were synthesized by GL Biochem Ltd. (Shanghai, China), and then were further purified and identified as described in our previous report (Chai et al., 2021).

### *P. acnes* Proliferation Inhibition Analysis

Minimum inhibitory concentration (MIC) and minimum bactericidal concentration (MBC) of Cath-MH against *P. acnes* ATCC 6919 and ATCC 11827 were determined using two-fold broth dilution method as previously described by us (Ye et al., 2020). Specifically, two strains *P. acnes* ATCC 6919 and ATCC 11827 acquired from Guangdong Institute of Microbiology were grown in brain heart infusion (BHI) broth (HKM, China) under MGC Anaeropack systems (Mitsubishi, Gas Chemical, Japan) which provides anaerobic conditions. A two-fold serial dilution of peptide was added to 96-well plate (Costar, Corning, United States) at final concentrations of 0.78, 1.56, 3.13, 6.25, 12.5, 25, and 50 μM before an equal volume of bacteria in fresh BHI broth (the microbial loading was 10^6^ CFU/ml) was loaded. After 72 h incubation at 37°C, the absorbance measurement of *P. acnes* suspension solution was done at 600 nm with microplate reader (Infinite M1000 Pro, Tecan Company, Switzerland). As a positive control, clindamycin (Sigma, United States) was used. MIC was defined as the minimum concentration inhibiting visible growth. To determine the minimum concentration of Cath-MH causing bacterial death, MBC was then determined following the MIC assay. 10 μl of sample which exhibited no evident growth after 72 h incubation was inoculated onto BHI agar plates. These plates were placed at 37°C for another 72 h under an anaerobic atmosphere. The MBC was defined as the peptide concentration at which there was no colony growth (Andrä et al., 2005).

### Bacterial Killing Kinetic Assay

The bacterial killing kinetics of Cath-MH against *P. acnes* ATCC 6919 were carried out according to our previous method with minor modification (Ye et al., 2020). Briefly, Cath-MH at final concentration of 1× MIC was mixed with an equal volume of bacteria in fresh BHI broth under an anaerobic atmosphere. Duplicate samples were withdrawn at various timepoints (0, 15, 30, 60, 90, 120, 150, and 180 min) and spread on BHI agar plates. The 0 timepoint represents the sample withdrawn immediately after mixing. Viable colonies were counted after incubation of the plates for 72 h at 37°C under anaerobic conditions. Clindamycin at 1× MIC value and sterile saline were applied as the positive and negative control, respectively.

### Bacterial Agglutination Test

The bacterial agglutination assay was done according to the method previously reported by us (Wu et al., 2021). In brief, *P. acnes* ATCC 6919 at exponential phase were harvested, washed twice and diluted to 2.0 × 10^8^ CFU/ml of density with fresh BHI broth and incubated with BSA, Cath-MH (2× MICs), or Cath-MH (2× MICs) plus equal volume of 0.2 mg/ml LPS (L2880, *Escherichia coli* O55:B5, Sigma, United States) or 0.2 mg/ml LTA (L2512, *Staphylococcus aureus*, Sigma, United States) at 37°C for 30 min. The mixture was dropped on a glass slide and dyed with a Gram staining kit (Solarbio Technology, Beijing), and the results were observed under an oil microscope (Nikon Corporation, Japan).

### Circular Dichroism Measurement

The Circular Dichroism (CD) measurement was performed to study the interaction of Cath-MH with LTA or LPS. In brief, Cath-MH was prepared in H_2_O or 30 mM SDS solution. Then, LTA or LPS (0.2 mg/ml) was loaded to the peptide solution (50 μM) for 1 h at room temperature, respectively. Binding of Cath-MH to LTA or LPS was studied by monitoring the change in its secondary structure. CD measurement was then carried out with Jasco-810 spectropolarimeter (Jasco, Japan). CD data were presented as the mean residue ellipticity (θ) of three consecutive scans per sample in deg·cm^2^·dmol^−1^.

### LTA Binding Assay

LTA binding of Cath-MH was further confirmed by measuring the inhibitory effects of LTA on the antimicrobial activity of Cath-MH against *P. acnes* ATCC 6919. In detail, LTA at concentrations (0, 0.0625, 0.125, 0.25, and 0.5 mg/ml) dissolved in sterile saline was mixed with 0.5, 1, and 2× MICs of Cath-MH for 30 min. Then, an equal volume of 10^6^ CFU/ml bacterial suspension in fresh BHI broth was added to the above mixture before coated on BHI agar plates. After anaerobic incubation for 72 h, the number of colonies were calculated. All experiments were repeated three times.

### Membrane Permeability and Morphology Alteration Analysis

To ensure the underlying mechanism of action of Cath-MH against *P. acnes* ATCC 6919, confocal laser scanning microscopy (CLSM) and scanning electron microscopy (SEM) experiments were performed for determination of the membrane permeability and morphological changes of *P. acnes*. Briefly, *P. acnes* ATCC 6919 at logarithmic growth phase were diluted to 10^6^ CFU/ml and incubated with Cath-MH (2× MICs) for 30 min at 37°C. SYTO9 and PI staining (LIVE/DEAD^®^ BacLight kit, Invitrogen, USA) was added to the bacterial suspensions, followed by incubation for 30 min at room temperature in the dark. CLSM (Leica TCS SP5, Leica Microsystems, Germany) was used to detect SYTO9 and PI with the excitation/emission spectrum of 480 and 635 nm, respectively. For SEM observation, bacterial suspensions at logarithmic growth phase were incubated with Cath-MH (2× MICs) for 30 min at 37°C. Then, *P. acnes* were harvested by centrifugation, sequentially fixed with 4 and 2.5% glutaraldehyde solution at room temperature for 4 h and 2.5% at 4°C overnight, respectively. After three times of wash with PBS, bacteria were dehydrated sequentially with 30, 50, 70, 85, 90, and 100% ethanol solution, followed by tert-Butyl alcohol, and dried in a freeze dryer (Quorum, UK). After gold coating, bacterial morphology was visualized by JSM-840 instrument (Hitachi, Japan) at the magnification of ×50,000.

### Cytotoxic Analysis

The cytotoxicity of Cath-MH on RAW 264.7 cells was measured by the MTT method as reported previously by us (Zeng et al., 2020). In short, RAW 264.7 cells at a density of 5,000 cells per well were plated in 96-well plates and grown with medium DMEM in the presence or absence of continuous concentrations of Cath-MH (2.5, 5, 10, 20, and 40 μM) at 37°C for 24 h before MTT was added in the dark and the culture was continued for another 4 h. The supernatant was discarded and DMSO was loaded before the absorbance at 490 nm as measured. The experiment was repeated at least three times.

### Membrane Binding Assays

Membrane binding assays were undertaken with FITC-labeled Cath-MH. In short, RAW 264.7 murine macrophage cells in the logarithmic phase of growth were prepared in PBS at a density of 1 × 10^5^ cells/ml and incubation with FITC-labeled Cath-MH (0, 2, 4, 8, and 16 μM) at 37°C for 30 min. The unbound peptide was washed out with PBS containing 1% BSA in advance. Cell fluorescence intensity was detected with a FACscan flow cytometer (Becton Dickinson, United States), representing the ability to bind to cell membranes. Cells without peptide treatment were regarded as the negative control.

### NO and Pro-Inflammatory Cytokine Measurement

RAW 264.7 murine macrophage cells at the density of 1 × 10^5^ cells per well were added into 24-well plates and grown for 12 h for adherence. The cells were pretreated with Cath-MH (0, 1, 2, 4, and 8 μM) for 1 h and then co-incubated with LTA (10 μg/ml) or LPS (100 ng/ml) for 24 h. Then, the culture supernatants were used for analysis of NO production by Griess reagent (Beyotime Biotechnology, China) and IL-1β, IL-6, and TNF-α levels using enzyme linked immunosorbent assay (ELISA) (Thermo Fisher Scientific, United States) in light of the manufacturer’s manuals.

### Quantitative Real-Time PCR

RAW 264.7 murine macrophage cells at the density of 1 × 10^6^ cells per well were added into 6-well plates and cultured for 12 h for adherence. The cells were pretreated with Cath-MH (0, 1, 2, 4, and 8 μM) for 1 h and then stimulated with LTA (10 μg/ml) or LPS (100 ng/ml) for 6 h at 37°C in 5% CO_2_. Cells were subsequently harvested to measure the mRNA levels of iNOS, IL-1β, IL-6, TNF-α, TLR2, and TLR4 by qRT-PCR as reported previously by Zeng (Zeng et al., 2018). GAPDH gene was applied as a control to standardize the amount of the sample mRNA. Forty amplification cycles were required to complete exponential amplification.

### Western Blot Analysis

RAW 264.7 murine macrophage cells were plated in 6-well plates at the density of 1 × 10^6^ cells/well and grown for 12 h for adherence. The cells were pretreated with Cath-MH (0, 1, 2, 4, and 8 μM) for 1 h and then stimulated with LTA (10 μg/ml) or LPS (100 ng/ml) at 37°C for 30 min in 5% CO_2_. After that, the cells were lysed with RIPA lysis buffer (Beyotime Biotechnology, China) and proteins were extracted using commercial kit (Cayman Chemical, United States) in light of the manufacturer’s recommendations. Primary antibodies of phospho-ERK/ERK, phospho-JNK/JNK, phosphor-p38/p38, NF-κB p65, Lamin A/C and GAPDH (1: 1,500, Cell Signaling Technology, United States) and horseradish peroxidase conjugated secondary antibodies (1: 2,000, Cell Signaling Technology, United States) were applied in western blot analysis. All experiments were repeated three times.

### *In vivo* Anti-Acne Analysis

The *in vivo* anti-acne effect of Cath-MH was evaluated using the procedure described previously by us (Ye et al., 2020). Six-week-old BALB/c mice weighing about 22 g were randomly subdivided into four groups (*n* = 6). Approximately 25 μl of *P. acnes* (5 × 10^8^ CFU/ml) was intradermally administrated into the left ears of mice and the control mice received an equivalent volume of PBS. Clindamycin (10 μg) and Cath-MH (50 μg) were mixed in 50 mg of sterile vaseline and then were painted onto the ear surfaces, respectively. The ear thickness was measured at 24 h with a micro-caliper (Mitutoyo, Japan) after *P. acnes* injection. Afterwards, mice were sacrificed and ears were sampled for bacterial cell counts, histopathological assay, ELISA, qRT-PCR, and western blot detection.

### Statistical Analysis

qRT-PCR data were calculated with the 2^−ΔΔCT^ method. All data were expressed as mean ± SEM. Statistical analysis were carried out using one-way ANOVA. *p < 0.05, **p < 0.01, and ***p < 0.001 were considered statistically significant as compared to control.

## Results

### Anti-*P. acnes* Activity

The effect of Cath-MH on *P. acnes* was determined using MIC and MBC assays. As shown in Table 1, both MIC and MBC values of Cath-MH against *P. acnes* ATCC 6919 and ATCC 11827 were about 1.56 μM. However, the MICs of clindamycin against two stains were about 3.39 μM. Moreover, its MBCs against *P. acnes* ATCC 6919 and ATCC 11827 were approximately 6.77 and 13.54 μM, respectively. Therefore, Cath-MH has more potent anti-*P. acnes* activity than the positive control clindamycin.

**TABLE 1:** Anti-*P. acnes* activity of Cath-MH.

Microorganisms	MIC (μM)		MBC (μM)	
	Cath-MH	Clindamycin	Cath-MH	Clindamycin
*P. acnes* ATCC 6919	1.56	3.39	1.56	6.77
*P. acnes* ATCC 11827	1.56	3.39	1.56	13.54

**FIGURE 1:**
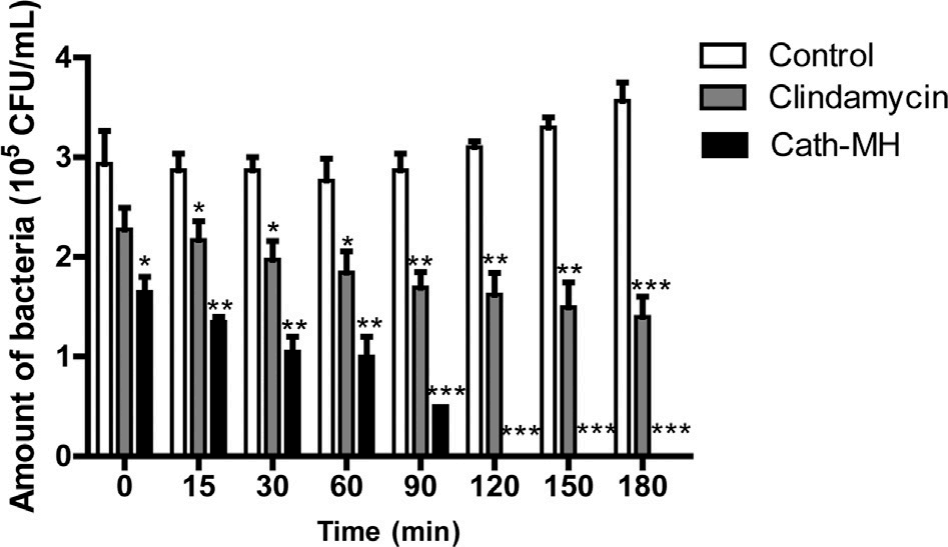
Killing kinetics of Cath-MH against *P. acnes* ATCC 6919. Bacteria were treated with Cath-MH at their 1× MIC of concentration for different time. Clindamycin was used as the positive control. Data are represented as mean ± SEM (*n* = 3) *p < 0.05, **p < 0.01, and ***p < 0.001 were considered statistically significant as compared to the corresponding control groups incubated with sterile saline for different time.

To further explore the anti-*P. acnes* activity of Cath-MH, its bacterial killing kinetics against *P. acnes* ATCC 6919 was evaluated. As seen in Figure 1, Cath-MH (1× MIC) exhibited potent bactericidal activity within 120 min of incubation. However, under same circumstances, clindamycin (1× MIC) showed much slower killing kinetic, which could not completely kill bacteria in 180 min.

### *P. acnes* Agglutination and LTA/LPS Binding Activities

**FIGURE 2:**
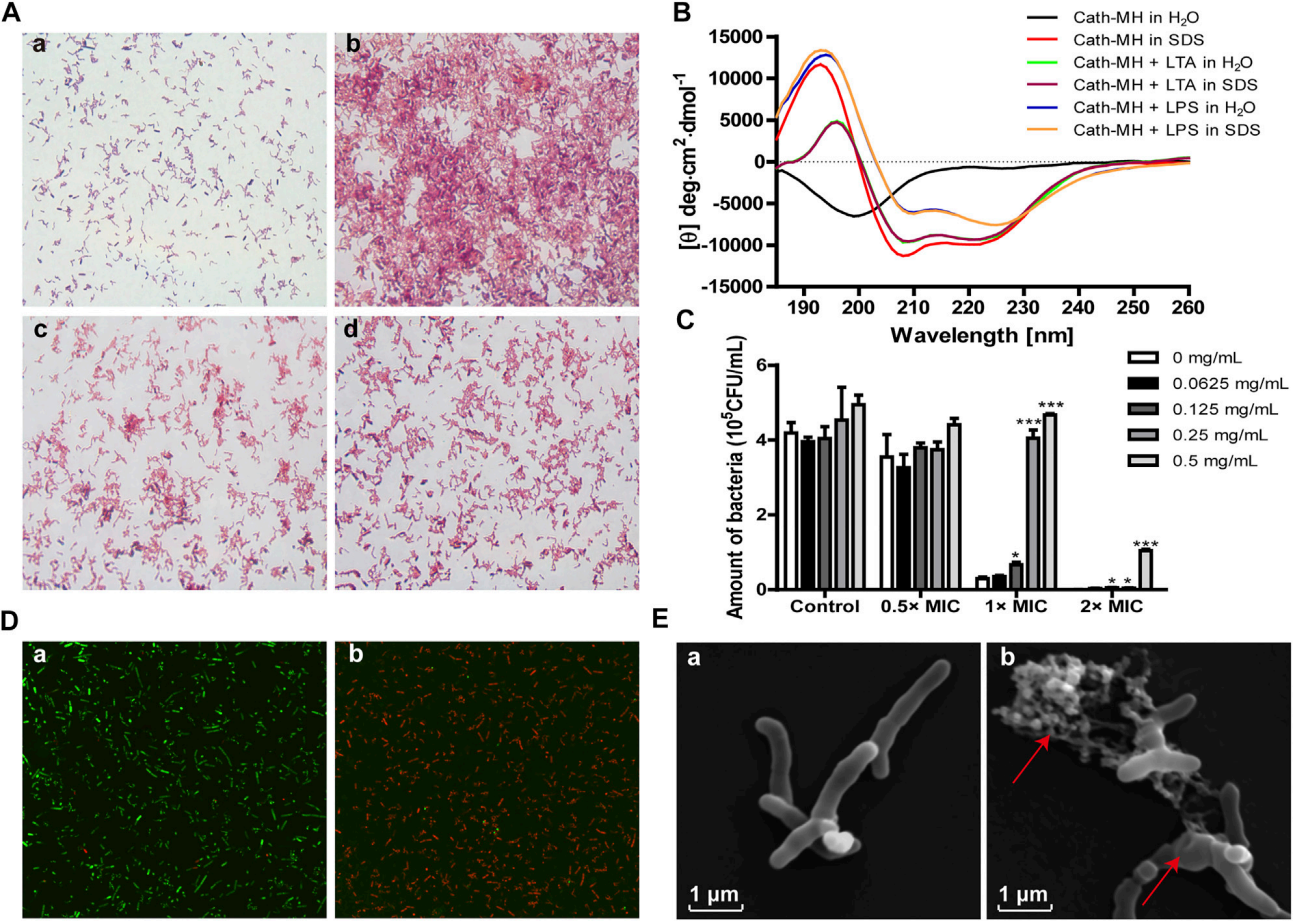
Anti-*P. acnes* mechanism of Cath-MH. **(A)** Agglutination of *P. acnes* ATCC 6919 by Cath-MH. *P. acnes* were incubated with BSA (a), Cath-MH (2× MICs) (b), and Cath-MH (2× MICs) plus equal volume of 0.2 mg/ml LPS (c) or 0.2 mg/ml LTA (d) for 30 min before being stained with Gram staining kit. **(B)** CD spectra of 50 μM Cath-MH in different solutions. **(C)** Suppression of LTA on the anti-*P. acnes* activity of Cath-MH. Cath-MH at the concentrations of 0.5×, 1× and 2× MICs were mixed with indicated concentrations of LTA for 30 min and then incubated with *P. acnes* for 72 h before its MIC was measured. **(D)** CLSM observation. *P. acnes* treated with sterile saline (a), or Cath-MH (2× MICs, b) for 30 min, respectively. **(E)** SEM observation. *P. acnes* treated with sterile saline (a), or Cath-MH (2× MICs, b) for 30 min, respectively. Data are expressed as mean ± SEM (*n* = 3). *p < 0.05, ***p < 0.001 were considered statistically significant compared with the control group without LTA.

Cath-MH can agglutinate *E. coli* ATCC 25922 (Chai et al., 2021). Therefore, we measured its agglutination activity against *P. acnes* ATCC 6919. As shown in Figure 2A, Cath-MH displayed a high agglutinating activity after incubation with *P. acnes* for 30 min (panel b) when compared with the control treated with BSA (panel a). However, this agglutination was abolished by 0.2 mg/ml LPS (panel c) and 0.2 mg/ml LTA (panel d), respectively, indicating that Cath-MH could bind LPS and LTA.

The LTA/LPS-binding ability of Cath-MH was further supported by CD spectroscopy and antimicrobial assay. As presented in Figure 2B, in the presence of LTA or LPS, Cath-MH showed obviously different CD spectra which were similar to peptide dissolved in SDS solution. Furtherly, the suppression effects of increasing concentrations of LTA on antimicrobial activities of Cath-MH at 0.5, 1, and 2× MICs against *P. acnes* were examined. As expected, LTA alone did not change the proliferation of *P. acnes*. However, the anti-*P. acnes* activities of Cath-MH at 1× and 2× MICs were significantly decreased after incubation with LTA at concentrations ranging from 0.0625 mg/ml to 0.5 mg/ml for 30 min. Moreover, this inhibitory effect depended on the concentration of LTA (Figure 2C).

### Effects on the Cell Membrane of *P. acnes*

AMP generally starts their killing bacteria process promptly once their attraction and attachment to microbial surfaces (Vineeth Kumar and Sanil, 2017). CLSM was carried to ensure the permeabilization of bacteria caused by Cath-MH. The results showed the Cath-MH-treated group with intense red fluorescence, which confirmed the majority of *P. acnes* with damaged membranes, while the untreated group showed intense green fluorescence which indicated with intact cell membrane (Figure 2D). To better understand the mechanism of action of Cath-MH against *P. acnes*, SEM was used. As shown in Figure 2E, after Cath-MH treatment, *P. acnes* had changed in morphology including obviously wrinkling and cell contents releasing (areas indicated by arrow) compared to the control group. Altogether, these data demonstrated that the Cath-MH treatment could effectively destroy the bacterial cell membrane and then kill bacteria, like most AMPs.

### Suppression of Inflammatory Factor and TLR2/4 Expression Induced by LTA/LPS

**FIGURE 3:**
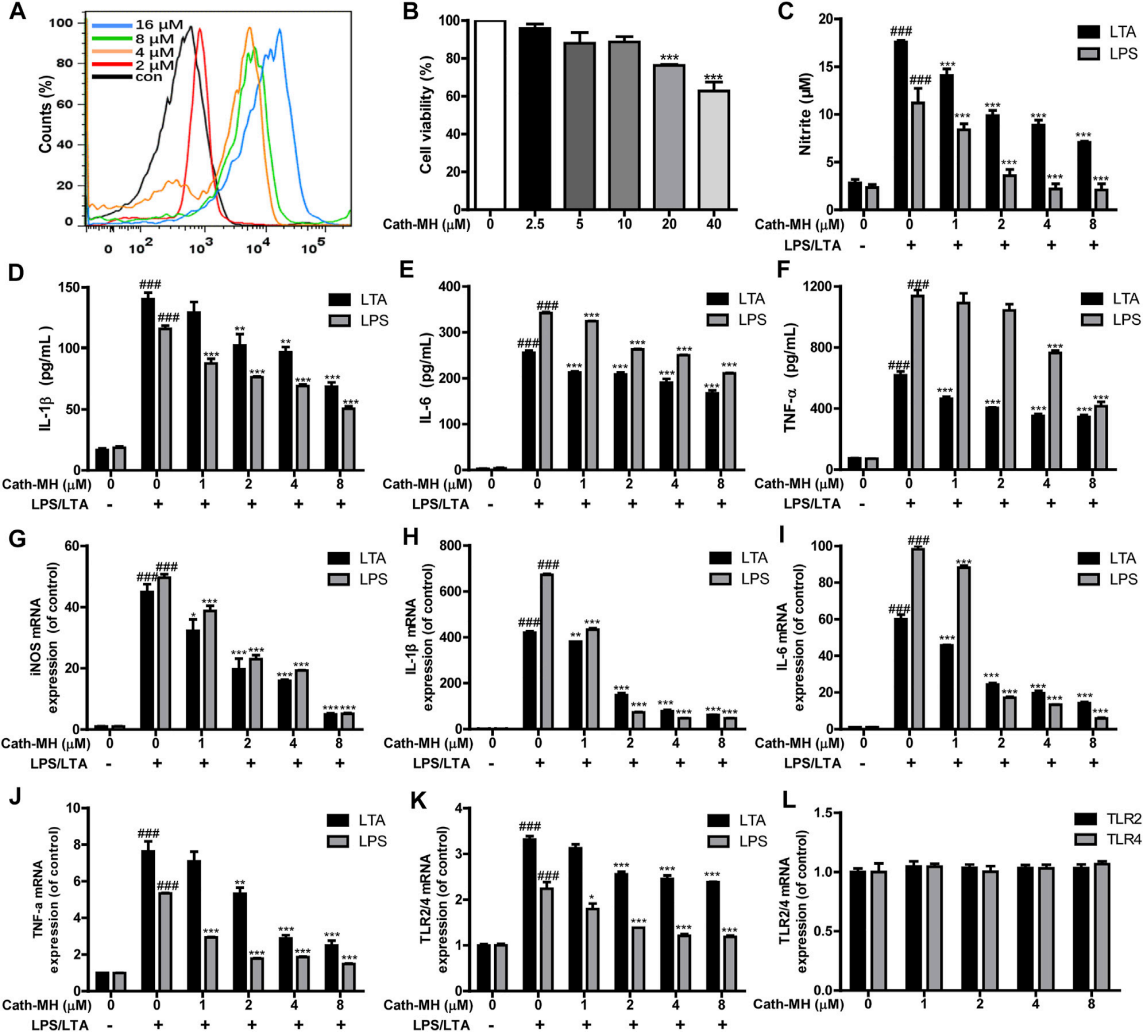
Suppression effects of Cath-MH on inflammatory cytokine and TLR2/4 expression stimulated by LTA or LPS. **(A)** Flow cytometry analysis of interaction between FITC-labeled Cath-MH and RAW 264.7 cells. **(B)** Cytotoxicity of Cath-MH towards RAW 264.7 cells. **(C–F)** Effects of Cath-MH on nitrite **(C)**, IL-1β **(D)**, IL-6 **(E)**, and TNF-α **(F)** levels stimulated by LTA or LPS. **(G–J)** Effects of Cath-MH on the mRNA levels of iNOS **(G)**, IL-1β **(H)**, IL-6 **(I)**, and TNF-α **(J)**. **(K,L)** Effects of Cath-MH on the mRNA levels of TLR2/4 induced by LTA/LPS **(K)** or without LTA/LPS stimulation **(L)**. Data are reported as mean ± SEM (*n* = 3). ^###^ *p* < 0.001, significantly different compared with the control group without LTA/LPS and Cath-MH; *p < 0.05, **p < 0.01, ***p < 0.001 were significantly different compared with the control group with LTA/LPS but without Cath-MH treatment.

Some AMPs can bind to their targets on the surface of macrophages, and then set off cellular signaling pathway and regulate the secretion of pro-inflammatory factors (Wang et al., 2011; Wei et al., 2015; Magrone et al., 2018; Zeng et al., 2018; Kasus-Jacobi et al., 2020; Chai et al., 2021). Therefore, the binding of Cath-MH to RAW 264.7 cells were evaluated with flow cytometry. As displayed in Figure 3A, Cath-MH could concentration-dependently bind to RAW 264.7 cells after co-incubation for 30 min. To define whether Cath-MH can affect the release of inflammatory factors in RAW 264.7 cells stimulated by LTA/LPS, we tested firstly its effect on the viability of RAW 264.7 cells. As shown in Figure 3B, Cath-MH at the concentrations of less than and equal to 10 μM had no cytotoxicity toward RAW 264.7 cells. Thus, we further investigated whether Cath-MH at the concentration of lower than 10 μM could inhibit the generation of inflammatory factors induced by LPS/LTA. As shown in Figures 3C–J, both 100 ng/ml LPS and 10 μg/ml LTA significantly increased the protein and mRNA contents of NO, IL-1β, IL-6, and TNF-α in the cell culture supernatants in comparison with the control without any treatment. However, the enhanced expressions induced by LPS/LTA were markedly reversed by Cath-MH in concentration-dependent manner. LTA/LPS as the TLR2/4 agonist can induce the TLR2/4-mediated inflammatory response and the expression elevation of TLR2/4 at mRNA and protein levels, respectively (Kwak et al., 2015). Therefore, we examined whether or not the suppressive effects of Cath-MH on LTA/LPS-induced cytokine production was correlated to TLR2/4 expression. The results showed that, with Cath-MH pre-treatment, TLR2/4 mRNA expression induced by LTA/LPS was downregulated (Figure 3K). However, without LTA/LPS stimulation, Cath-MH did not change TLR2/4 mRNA expression (Figure 3L), suggesting that the effect of Cath-MH on inflammatory factor expressions is associated with its suppression of TLR2/4 mRNA expression stimulated by LTA/LPS.

### Inhibition of LPS-Activated Inflammatory Response Pathways

**FIGURE 4:**
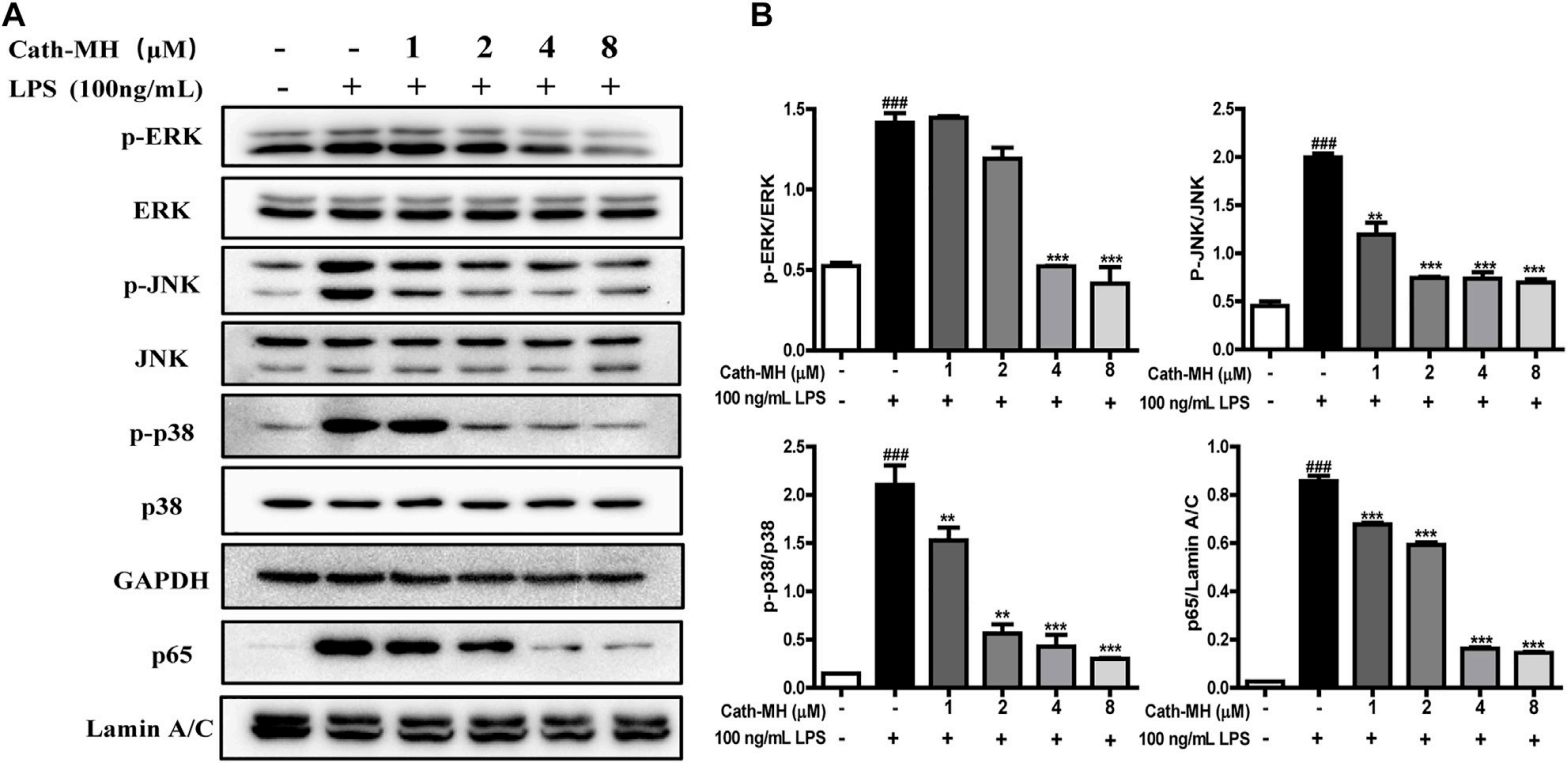
Effects of Cath-MH on inflammatory response pathways in LPS-activated RAW 264.7 cells. **(A)** Western blot of ERK, JNK, p38, and NF-κB p65 in LPS-stimulated RAW 264.7 cells. GAPDH were served as the internal control and Lamin A/C served as the nuclear control. **(B)** Ratios of phosphorylated ERK, JNK, p38 to corresponding total protein and NF-κB p65 to Lamin A/C, respectively. Band densities were analyzed using ImageJ software (NIH Image, United States). Data are reported as mean ± SEM (*n* = 3). ^###^ *p* < 0.001 significantly different compared with the control group without LPS and Cath-MH; **p < 0.01, ***p < 0.001, significantly different compared to the control group with LPS but without Cath-MH treatment.

It has been well known that MAPK/NF-κB signaling pathways play a vital role in pro-inflammatory process by regulating the expression of inflammatory factors including IL-1β, IL-6, and TNF-α. Therefore, we evaluated the effects of Cath-MH on MAPK/NF-κB signaling pathways in LPS-stimulated RAW 264.7 cells using western blot. As presented in Figure 4, LPS (100 ng/ml) significantly increased the expression of phosphorylated ERK, JNK, p38, and the nuclear translocation of NF-κB p65 when compared to the control group. However, this upregulation induced by LPS was significantly repressed by Cath-MH in a dose-dependent manner.

### Anti-Acne Effects *In Vivo*

**FIGURE 5:**
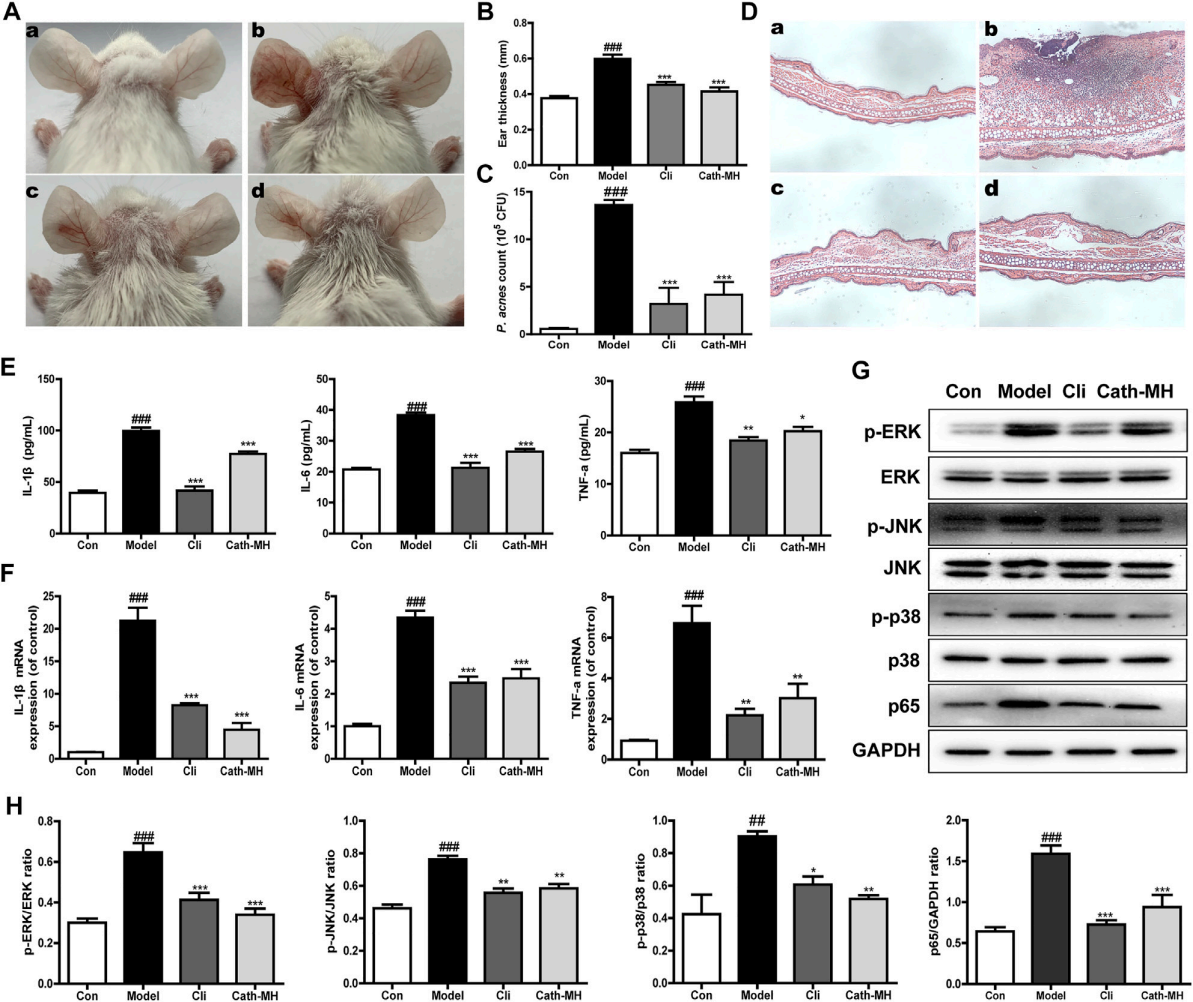
Effects of Cath-MH on acne vulgaris in mice. **(A)** Representative ear photographs after *P. acnes* exposure for 24 h. **(B)** Statistical analysis of mice ear thickness. **(C)** Statistical analysis of the counts of *P. acnes* in mice ear. **(D)** Histopathological analysis of mouse ear. **(E,F)** The protein and mRNA levels of IL-1β, IL-6, and TNF-α in mouse ear. **(G)** Western blot of ERK, JNK, p38, and NF-κB p65 in mouse ear. **(H)** Ratio of phosphorylated ERK, JNK, p38, and NF-κB p65 to total protein. In **(A)** and **(D)**, panels a–d represent images captured from the mouse ear administrated by sterile saline (a), *P. acnes* (b), 10 μg of clindamycin (c) and 50 μg of Cath-MH (d) after injection of *P. acnes*, respectively. In column and WB graphs, con, model, cil and Cath-MH represent the statistical data in control, model, clindamycin- and Cath-MH-treated groups which are presented as the mean ± SEM (*n* = 5). ^###^ *p* < 0.001 significantly different compared to the control group; *p < 0.05, **p < 0.01, ***p < 0.001 significantly different compared to the model group.

The *in vivo* anti-acne activity of Cath-MH was explored using acne mouse model. As shown in Figure 5, following *P. acnes* injection, the ears of mice became red and swollen. However, like clindamycin, Cath-MH obviously relieved *P. acnes*-induced ear redness and swelling (Figures 5A,B). Moreover, as shown in Figure 5C, both Cath-MH and clindamycin substantially decreased the number of *P. acnes* colonized in the ear when compared with the model group only injected by *P. acnes* suspensions. To explore its effects on inflammation induced by *P. acnes,* histopathological analysis and cytokine expression measurement were also performed. As shown in Figure 5D **,** the infiltration of inflammatory cell and ear swelling were obviously increased in the ear injection of *P. acnes* when compared with the control sample. Nevertheless, it was significantly decreased after Cath-MH or clindamycin treatment. Consistently, following 24 h injection of *P. acnes*, both protein and mRNA expressions of TNF-α, IL-1β and IL-6 were all significantly upregulated. Yet, their upregulations were markedly reversed after treatment with Cath-MH or clindamycin (Figures 5E,F). To further clear the protective mechanism of Cath-MH in acne vulgaris mice, MAPK/NF-κB signaling pathways were investigated by western blotting (Figures 5G,H). Consistent with their effects on cytokine expression *in vitro*, injection of *P. acnes* significantly upregulated the expression of phosphorylated ERK, JNK, p38 and p65 translocated in nucleus of ear tissues when compared with vehicle. However, treatment with Cath-MH and clindamycin successfully suppressed these increases but had no influence on total ERK, JNK, and p38 expression. Together, these data demonstrated that the Cath-MH treatment can effectively improve acne vulgaris in mice.

## Discussion

Acne vulgaris is a chronic inflammatory conditions of the skin, concerning colonization of *P. acnes*, and subsequent activation of immune cells (Vowels et al., 1995), then ensuing excessive secretion of pro-inflammatory cytokines such as IL-1β, IL-6 and TNF-α which in turn results in inflammatory cascade and tissue injury (Vowels et al., 1995; Dréno, 2017). Therefore, it is generally considered as promising treatment for acne vulgaris to inhibit colonization of *P. acnes* and harmful inflammatory response (Cong et al., 2019; Habeshian and Cohen, 2020). Several AMPs such as Esc-1GN, LZ1, and Cathelicidin-BF are reported to show potent treatment potential for Acne vulgaris because of their suppressive effects on *P. acnes* and inflammation (Wang et al., 2011; Zhang et al., 2013; Ye et al., 2020). In our study, the overwhelming evidences have confirmed that Cath-MH can kill and agglutinate *P. acnes*, bind LTA/LPS, and suppress inflammation induced by *P. acnes in vitro* and *in vivo*. Furtherly, Cath-MH can more strongly and quickly inhibit the growth of the tested *P. acnes in vitro* than clindamycin which is an antibiotic frequently used to treat acne vulgaris (Figure 1; Table 1). Secondly, agglutination of *P. acnes by* Cath-MH prevents dissemination of the infection focus and facilitates the infection clearance by the host innate cells (Figure 2A). Thirdly, it makes less likely to induce bacterial resistance that Cath-MH directly kills *P. acnes* by the membrane disruption mechanism (Figures 2D,E) (Yeaman and Yount, 2003). Finally, Cath-MH is relatively low cytotoxicity to mammal cells and high stability *in vitro* and *in vivo* (Chai et al., 2021). Taken together, these findings suggest that Cath-MH with anti-*P. acnes* and anti-inflammatory activity is an excellent candidate drug molecule against acne.

The engagements of TLR4 by LPS and TLR2 by LTA trigger the activation of downstream intracellular NF-κB/MAPK signaling pathways, consequently causing the generation of pro-inflammatory mediators such as TNF-α, IL-1β and IL-6 (Wu et al., 2017). Human LL-37 and chicken CATH-2 have been reported to suppress TLR2/4 activation by directly interaction with the outer membrane-derived lipoproteins and LPS (Coorens et al., 2017). In addition, cationic peptide P5 suppresses TLR2-to-NF-κB signaling by binding to LTA, thereby inhibiting the production of inflammatory factors (Ryu et al., 2015). Considering that Cath-MH can bind to LPS (Chai et al., 2021) as well as LTA (Figures 2A–C), we conjectured that Cath-MH has anti-inflammatory effects related to TLR2/4. Macrophages as crucial immune cells are involved in the regulation of many chronic inflammatory diseases including Acne vulgaris through the secretion of a great deal of pro-inflammatory cytokines and chemokines (Kim, 2005; Navegantes et al., 2017), and are extensively used as an *in vitro* inflammation model assessing the potential protection of a drug (Lee et al., 2020; Nguyen et al., 2020). Thus, in this study, we used RAW 264.7 murine macrophage cells to identify the anti-inflammation effects and underlying mechanism of Cath-MH (Zhao et al., 2017). In agreement with our assumption, Cath-MH is found to significantly inhibit the TLR2/TLR4 expression, inflammatory factor secretion and MAPK/NF-κB pathway activation in RAW 264.7 cells stimulated by LPS/LTA (Figures 3, 4). Cath-MH also produces similar effects in mouse ears with exposure to *P. acnes* (Figure 5). It has been described that both *P. acnes* and its important component, LTA, can induce not only TLR2/4 expression but also pro-inflammatory cytokine release associated with TLR2/4 signaling pathways *in vitro* and *in vivo* (Jugeau et al., 2005; Kwak et al., 2015; Dréno, 2017; Suvanprakorn et al., 2019). Therefore, Cath-MH may have anti-inflammatory effects by binding LPS/LTA and blocking TLR2/4-mediated MAPK/NF-κB signaling pathways in macrophage cells and acne mouse models.

Some AMPs like Hc-CATH and HPA_3_NT_3_ have been reported to, respectively, bind TLR4 or TLR2, accordingly suppressing the activation of TLR2/4-mediated signaling pathways (Ni et al., 2014; Ryu et al., 2014; Wei et al., 2015). As shown in Figure 3A, Cath-MH can bind directly to the surface of RAW 264.7 cells. Additionally, Cath-MH can also reduce carrageenan-stimulated inflammation in mouse paw in the absence of LPS and LTA (Data not shown). Thus, it is possible that Cath-MH bind its receptors on the membranes of macrophages, consequently inhibiting the activation of MAPK/NF-κB pathways and the transcription of NO and other inflammatory cytokines *in vitro* and *in vivo*. Therefore, further study is necessary to explore its anti-inflammatory mechanism of Cath-MH. Finally, our previous studies have showed that Cath-MH reduces the LPS-induced inflammation in sepsis (Chai et al., 2021). In the present study, Cath-MH attenuates LTA- and *P. acnes-*induced inflammation, which extends the scope of inflammatory agonists antagonized by Cath-MH and help to clarify its anti-inflammation mechanism.

In conclusion, we demonstrate that Cath-MH exerts direct antimicrobial effects against *P. acnes* by aggregation of bacterial cells and disruption of the bacterial cell membrane. Cath-MH also binds to LTA/LPS, inhibits LTA/LPS-stimulated TLR2/4 expression, and subsequently reduces the production of the inflammatory cytokines through blocking MAPK/NF-κB signaling pathways *in vitro*. Consistently, Cath-MH displays both anti-*P. acnes* and anti-inflammatory effects in *P. acnes*-stimulated mouse model via inhibition of *P. acnes* proliferation, inflammatory cytokines expression as well as MAPK/NF-κB signaling activation. Taken together, these findings indicate that Cath-MH can potentially serve as an effective therapeutic agent for the treatment of acne vulgaris.

## Data Availability

The raw data supporting the conclusion of this article will be made available by the authors, without undue reservation.
